# The cost-effectiveness analysis of single-tablet efavirenz-based regimen among HIV-1 infected adults in China

**DOI:** 10.3389/fpubh.2025.1429461

**Published:** 2025-05-08

**Authors:** Haijun Zhang, Yuhao Kong, Haotian Chen, Sitong Luo, Pengyang Fan, Zhihui Li

**Affiliations:** ^1^Department of Health Policy and Management, School of Public Health, Peking University, Beijing, China; ^2^Department of International Health, Bloomberg School of Public Health, Johns Hopkins University, Baltimore, MD, United States; ^3^Vanke School of Public Health, Tsinghua University, Beijing, China; ^4^Institute for Healthy China, Tsinghua University, Beijing, China

**Keywords:** HIV, TLE regimen, cost-effectiveness, efavirenz, antiretroviral therapy

## Abstract

**Introduction:**

In 2018, the Chinese Guidelines for Diagnosis and Treatment of HIV/AIDS recommended the adoption of the efavirenz 400 mg-based TLE (tenofovir disoproxil fumarate (TDF) + lamivudine (3TC) + efavirenz (EFV)) regimen as the primary first-line treatment for ART-naive HIV-1 infected adults in China. However, the cost-effectiveness of different TLE treatment strategies remains uncertain. This study aimed to evaluate the cost-effectiveness of various TLE treatment strategies for ART-naive HIV-1 infected adults in China.

**Methods:**

A decision-tree Markov state transition model was employed to assess the cost-effectiveness of various TLE treatment strategies over a 10-year timeframe, from a societal perspective. Input parameters were obtained from published literature and publicly accessible information. Local data from the latest sources were used as input parameters whenever possible. The main outcome measure was the incremental costs per quality-adjusted life years (QALYs) gained. Sensitivity analyses were performed to investigate model uncertainties and determine break-even prices.

**Results:**

Compared to the multiple-tablet regimen (MTR) consisting of efavirenz 400 mg-based TLE (TLE400) and efavirenz 600 mg-based TLE (TLE600), the single-tablet regimen (STR) of TLE400 exhibited a 10-year cost of 130733.8 CNY (compared to 122939.7 CNY and 126184.3 CNY, respectively) and an expected QALYs of 6.45 (compared to 6.27 QALYs and 6.32 QALYs, respectively) per HIV-1 patient in China. Consequently, the incremental cost-effectiveness ratios (ICERs) were 41021.6 CNY/QALY gained (equivalent to US$ 6071.2 per QALY gained) and 34996.2 CNY/QALY gained (equivalent to US$ 5179.4 per QALY gained) for TLE400 STR compared to TLE400 MTR and TLE600 MTR, respectively. The ICER for TLE400 MTR compared to TLE600 MTR was 54076.7 CNY/QALY gained (equivalent to US$ 8003.4 per QALY gained). Deterministic sensitivity analysis indicated that adherence rates to ART had the most significant influence on all three strategies. In probabilistic sensitivity analysis, TLE400 STR demonstrated a 71.4% probability of being highly cost-effective nationwide, based on the one-time national-level GDP per capita.

**Conclusion:**

In the context of treating HIV-1 infected adults in China, the STR of TLE400 demonstrated cost-effectiveness when compared to both the MTR of TLE400 and the MTR of TLE600.

## Introduction

Human Immunodeficiency Virus/Acquired Immunodeficiency Syndrome (HIV/AIDS) is a chronic condition characterized by compromised immune function, resulting in a progressive decline in Cluster of Differentiation 4 (CD4) cell count and an increase in viral load. This leads to a gradual deterioration of the body’s ability to defend against infections (1). HIV/AIDS remains a significant global public health concern, attracting substantial attention from governments worldwide, including China (2). Since the identification of the first HIV case in China in 1985, the prevalence of HIV infection has steadily risen (3). According to the Chinese Center for Disease Control and Prevention (China CDC), as of the end of 2020, the number of individuals living with HIV in China reached 1.053 million, with a cumulative reported death toll of 351,000 (4). In 2021, HIV/AIDS-related deaths accounted for 19,623 cases, representing 88% of all infectious disease-related deaths (5).

In response to the escalating HIV burden, China has implemented various proactive strategies and interventions. One notable policy milestone is the National Free Antiretroviral Therapy Program (NFATRP) that commenced in 2003. This program offers cost-free diagnosis, counseling, treatment, and healthcare services to all individuals living with HIV. The NFATRP recommends the utilization of TLE therapy as the primary first-line antiretroviral therapy (ART) regimen, comprising tenofovir disoproxil fumarate (TDF), lamivudine (3TC), and efavirenz (EFV). However, prior studies have emphasized that the standard dosage of 600 mg EFV in the TLE regimen (TLE600) is associated with an increased occurrence of adverse effects, including abnormal liver function, dyslipidemia, depression, suicidal ideation, and reduced adherence rates (6–8).

Nonetheless, a pivotal study known as ENCORE1 has provided compelling evidence that an alternative regimen utilizing a lower dose of 400 mg EFV is non-inferior to the standard dose regimen of 600 mg EFV in treatment-naive patients, as demonstrated at both 48 and 96 weeks (9). Similarly, several noteworthy studies conducted in China have indicated that the 400 mg EFV regimen is associated with a reduced incidence of drug-related adverse events, decreased treatment discontinuation rates, and favorable safety profiles, including optimal virological and immunological efficacy, among adult patients (10–13). Based on these findings, the updated Chinese Guidelines for Diagnosis and Treatment of HIV/AIDS in 2021 have recommended the adoption of the 400 mg EFV-based TLE regimen (TLE400) as the primary first-line treatment for HIV-infected adults in China (14).

Based on evidence derived from previous trials, the TLE400 STR has been widely adopted in numerous developed countries (15–17). Comparative studies between the STR and conventional MTR have demonstrated superior adherence rates, reduced hospitalization, and increased patient satisfaction scores (18, 19). However, in the context of China, the TLE400 STR is not currently available, thereby creating a knowledge gap concerning its efficacy and safety within the Chinese population. Consequently, there is a need to assess whether this alternative regimen should be introduced to the Chinese population and to comprehensively understand its potential health economic implications. To address this gap and provide valuable insights into the potential trade-offs associated with the TLE400 STR, our study aimed to prospectively evaluate its cost-effectiveness for ART-naive HIV-1 infected adults in China.

## Methods

### Model design

**Figure 1:**
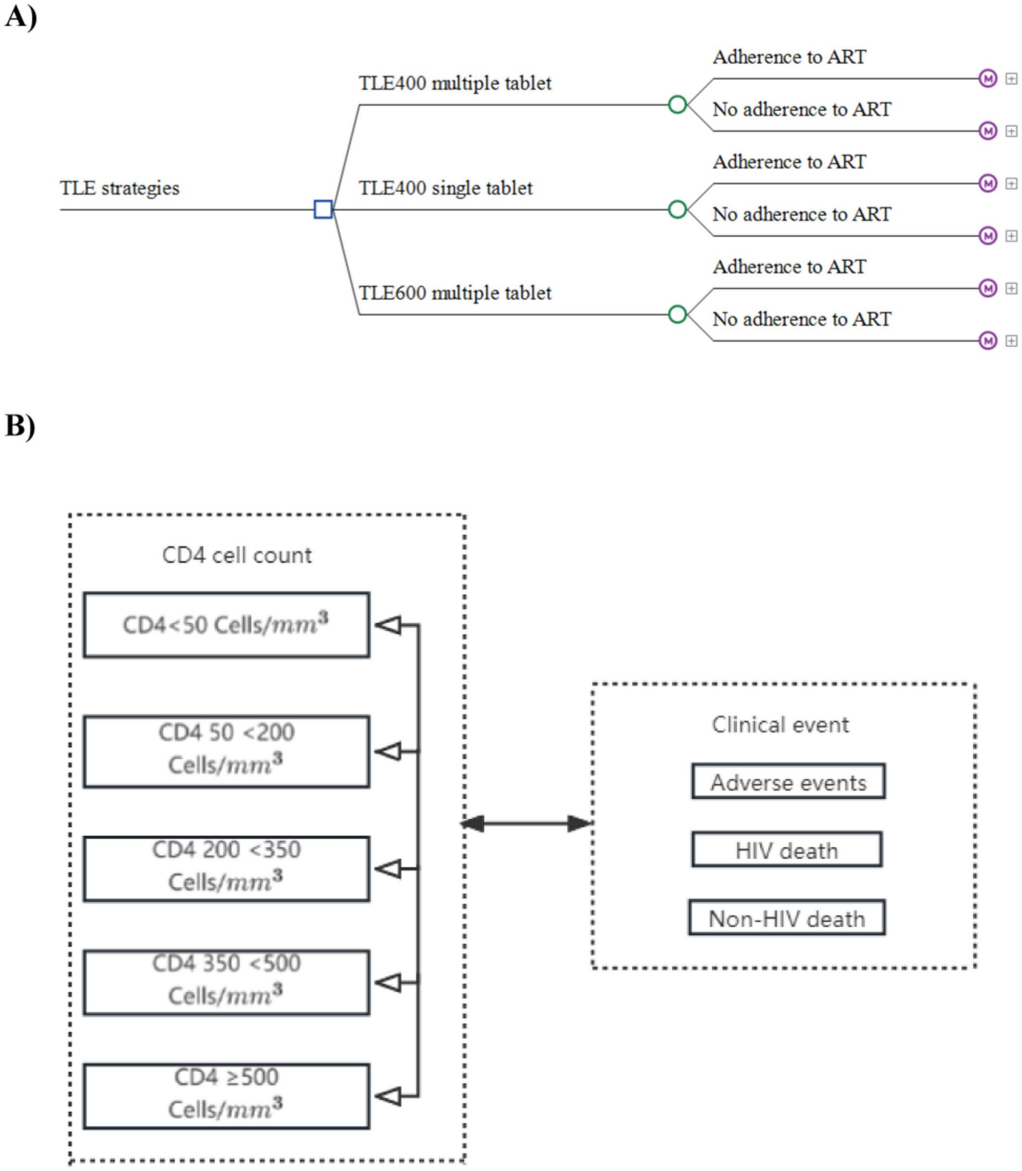
Markov decision tree for TLE400 single-tablet, TLE400 multiple-tablet, and TLE600 multiple-tablet regimen strategies. **(A)** Markov decision tree. **(B)** Markov diagram for HIV progression based on CD4 cell counts.

To evaluate and compare the clinical and economic performance of different treatment regimens, we utilized a decision-tree Markov state transition model in our study. The model focused on assessing the TLE400 STR, TLE400 MTR, and TLE600 MTR as first-line treatments for ART-naive HIV-1 infected adults (aged ≥18 years) in China. The decision tree component of the model comprised three branches representing the three treatment regimens: TLE400 single-tablet, TLE400 multiple-tablet, and TLE600 multiple-tablet (Figure 1A). Each branch was further divided into two strategies: adherence to ART and no adherence to ART. These strategies led to specific nodes in the Markov model. Based on existing research, we developed a Markov model that incorporated CD4 cell levels and clinical events, encompassing five HIV-infected states categorized by CD4 cell counts and three clinical events (Figure 1B). We selected CD4 cell counts as the outcome measure primarily based on data availability and comparability. The model operated in monthly cycles over a 10-year period, simulating disease-related costs and health outcomes for HIV-1 patients aged 18 and above in China.

The analyses in our study were conducted from a societal perspective, which takes into account both direct and indirect costs and effects. To ensure comparability, all costs were discounted to 2022 Chinese Yuan (exchange rate: 1 CNY = US$ 0.148) and adjusted for inflation as necessary (20). Health outcomes were assessed in terms of quality-adjusted life years (QALYs) gained, with the incremental costs per QALY gained serving as the primary outcome measure. Both costs and effectiveness were discounted at a rate of 5%, following the recommendations outlined in the China guidelines for pharmacoeconomic evaluations (21). Since specific cost-effectiveness thresholds for drugs in China were unavailable, we adopted one-time the 2022 national level GDP per capita (85698.0 CNY, equivalent to US $12683.3) as the threshold to evaluate the cost-effectiveness of the three treatment strategies (22). The model utilized in our analysis was developed using TreeAge Pro 2022 (TreeAge Software, Inc., Williamstown, MA).

### Model parameters and data sources

#### Clinical and epidemiological parameters

The clinical and epidemiological parameters utilized in our model were primarily obtained from large-scale randomized controlled trials, such as the ENCORE1 study, and domestic high-quality literature (9, 23). To ensure consistency and relevance, the trials and literature included subjects who met the following criteria: being over 18 years old, ART-naive, and non-pregnant. The initial CD4 count for individuals in the model was randomly determined using a gamma distribution, and an equation was developed to establish the upper limit of the CD4 count in the model. The baseline CD4 cell level for patients was assumed to be 352 cells/mm^3^ with a standard deviation of 209 cells/mm^3^, based on findings from a retrospective cohort study conducted in Henan province spanning over 14 years (24). The monthly increases in CD4 cell count in the model were derived from the ENCORE1 study, which involved patients receiving TLE400 and TLE600 regimens during different time intervals (0–48 weeks and 49–96 weeks) (9, 23). Following 96 weeks of the last ART treatment, based on a US observational study, it was assumed that patients would experience a decline in CD4 cell count (25, 26).

The monthly probabilities of treatment failure after initiation and the monthly decrease in CD4 cell count without treatment were obtained from relevant literature sources (27). Mortality rates for patients in different CD4 cell states were obtained from domestically published modeling research (28). The probabilities of severe adverse reactions for TLE400 and TLE600 were sourced from the ENCORE1 study (9, 23), while the rate of treatment interruption due to adverse reactions of EFV was derived from a systematic review study (29). Specific clinical and epidemiological parameters for each disease state during the model simulation can be found in Table 1.

**Table 1:** Model parameters and data sources.

Model parameter	Base case value	Ranges	Distribution	Sources
Clinical and epidemiological parameters				
CD4 cell count (cells/mm^3^) after 0–48 weeks of treatment (per month)				
TLE400	+16.9	15.6–18.2	Gamma	ENCORE1 Study (9)
TLE600	+14.6	13.5–15.8	Gamma	ENCORE1 Study (9)
CD4 cell count (cells/mm^3^) after 49–96 weeks of treatment (per month)				
TLE400	+4.5	4.2–4.7	Gamma	ENCORE1 Study (9)
TLE600	+4.4	4.2–4.7	Gamma	ENCORE1 Study (9)
CD4 cell count (cells/mm^3^) after 96 weeks of treatment (per month)	−2.32	±20%	Gamma	Tremblay et al. (40)
Probability of virological failure (per month)				
0–11 months	0.86%	±20%	Beta	Despiégel et al. (27)
12–22 months	0.08%	±20%	Beta	
After 22 months	0.06%	±20%	Beta	
CD4 cell count without treatment (per month)	−2.9	1.4–7.1	Gamma	Patrikar et al. (41)
Case fatality rate (per month)				
CD4 < 50 Cells/mm^3^	1.22%	±20%	Beta	Punekar et al. (28)
CD4 50 < 200 Cells/mm^3^	0.80%	±20%	Beta	Punekar et al. (28)
CD4 200 < 350 Cells/mm^3^	0.52%	±20%	Beta	Punekar et al. (28)
CD4 350 < 500 Cells/mm^3^	0.17%	±20%	Beta	Punekar et al. (28)
CD4 ≥ 500 Cells/mm^3^	0.07%	±20%	Beta	Punekar et al. (28)
Adverse events				
EFV 400 mg	13%	±20%	Beta	ENCORE1 Study (9)
EFV 600 mg	23%	±20%	Beta	ENCORE1 Study (9)
Probabilities of discontinuation due to adverse events	10%	9–16%	Beta	Patel et al. (29)
Adherence rates				
TLE-400 multi-tablets	77.5%	75–85%	Beta	Sutton et al. (30)
TLE-400 single-tablet	90%	85–95%	Beta	Sutton et al. (30)
TLE-600 multi-tablets	83.30%	80–90%	Beta	Xiao et al. (11)
Cost of drugs (CNY)				
TLE-400 multi-tablets	2.63	±20%	Gamma	China Government Procurement Network (42)
TLE-400 single-tablet	2.80	±20%	Gamma	Expert consultation
TLE-600 multi-tablets	3.07	±20%	Gamma	China Government Procurement Network (42)
Cost of illness (CNY)				
CD4 < 50 Cells/mm^3^	1782.98	±20%	Gamma	Ma et al. (32)
CD4 50 < 200 Cells/mm^3^	1782.98	±20%	Gamma	Ma et al. (32)
CD4 200 < 350 Cells/mm^3^	1737.33	±20%	Gamma	Ma et al. (32)
CD4 350 < 500 Cells/mm^3^	1635.68	±20%	Gamma	Ma et al. (32)
CD4 ≥ 500 Cells/mm^3^	1530.74	±20%	Gamma	Ma et al. (32)
Adverse events	1193.55	±20%	Gamma	Wang et al. (33)
Utilities				
CD4 < 50 Cells/mm^3^	0.822	±20%	Beta	Punekar et al. (28)
CD4 50 < 200 Cells/mm^3^	0.861	±20%	Beta	Punekar et al. (28)
CD4 200 < 350 Cells/mm^3^	0.886	±20%	Beta	Punekar et al. (28)
CD4 350 < 500 Cells/mm^3^	0.899	±20%	Beta	Punekar et al. (28)
CD4 ≥ 500 Cells/mm^3^	0.896	±20%	Beta	Punekar et al. (28)
Adverse events	0.817	±20%	Beta	Kauf et al. (35)
Other parameters				
Natural mortality rate	0.707%	±20%	Beta	China Health Statistics Yearbook 2021 (36)
Discount rate	5%	0–8%	Beta	China guidelines for pharmacoeconomic evaluations (21)
2022 GDP per capita (CNY)	85,698	NA	NA	China National Bureau of Statistics (22)

#### Adherence rates

Adherence rates are crucial for the successful implementation of antiretroviral therapy in HIV patients. In our study, the adherence rate for the TLE600 MTR was obtained from a randomized controlled trial conducted specifically with HIV patients in China (11). However, since the TLE400 STR is not currently available in the domestic market, empirical research on its adherence and direct comparative studies on adherence between the TLE400 STR and MTR are unavailable. To address this data gap, we assumed regarding adherence rates based on a study that compared adherence between single-tablet and multiple-tablet HIV regimens (30). The adherence rates utilized in our analysis were assigned to the TLE400 STR and MTR, respectively (as shown in Table 1).

#### Cost data

The cost data utilized in this study were obtained from multiple sources. The prices of the three TLE treatment strategies were mainly sourced from the Chinese government procurement network and expert consultations (31). Specifically, in 2022, the prices of three TLE treatment strategies were 2.63 CNY (TLE400 MTR), 2.80 CNY (TLE400 STR), and 3.07 CNY (TLE600 MTR), respectively. The drug price of the TLE400 STR was obtained through expert consultations, while the drug prices of the TLE400 MTR and TLE600 MTR were extracted from the Chinese government procurement network in 2022. The costs associated with HIV-infected states stratified by CD4 cell counts were derived from a large-scale field survey conducted by the China CDC in Henan Province in 2015 (32). The field survey encompassed various cost components, including direct medical costs and non-direct medical costs related to antiretroviral therapy. Direct medical costs included drug costs, pre-examination, CD4 cell testing, follow-up costs, outpatient costs, and hospitalization costs.

The non-direct medical costs considered in this study encompassed transportation costs, accompanying costs, nutrition costs, lost labor costs, and ART management costs. The cost of illness in 2015 was adjusted to 2022 using the Consumer Price Index (CPI) for the period from 2015 to 2022 to account for inflation. The cost of treating adverse reactions was obtained from a domestic literature focusing on the medical costs and economic evaluation of HIV patients in Shijiazhuang, Hebei province (33). This study provided detailed information on the treatment costs associated with adverse reactions in HIV patients receiving TLE strategies in China. To obtain national-level costs, all local costs were adjusted by applying the health expenditure as a weight. The total cost divided by CD4 cells is presented in Table 1.

#### Utilities

Due to the unavailability of utility weights specifically for the baseline population and utility scores for patients with HIV-1 infection in China, utility scores utilized in this study were obtained from published literature in other countries (34, 35). These utility scores were adapted to represent the health-related quality of life for individuals in different health states associated with HIV-1 infection. The specific parameters associated with the utility scores utilized in the model are presented in Table 1. These parameters were derived from relevant literature that assessed the health-related quality of life in HIV-1 patients. It is important to acknowledge that the utility scores may not directly apply to the Chinese population, as there may be cultural and contextual variations that can influence health-related quality of life. However, in the absence of country-specific data, these scores provide a reasonable estimation for the utility weights employed in the analysis. Table 1 shown detailed utility scores corresponding to each health state in the model.

### Other parameters

Our study incorporates several additional parameters, including natural mortality rates, discount rates, and GDP per capita in 2022. These parameters are crucial for the analysis and were obtained from reliable sources. The natural mortality rates were derived from the China Health Statistics Yearbook 2021 (36). Discount rates, which are applied to both costs and effectiveness, were determined based on the China guidelines for pharmacoeconomic evaluations (21). The GDP per capita in 2022, denoted in CNY, was obtained from the China National Bureau of Statistics (22).

### Sensitivity analyses

Deterministic sensitivity analyses (DSA) and probabilistic sensitivity analysis (PSA) were performed to test the robustness of the model results and to examine the uncertainty in the model. DSA was conducted to explore the sensitivity of the results to plausible variations in data inputs, where relevant parameters changed one at a time within the range of plausibility specified in Table 1. For parameters with unknown uncertainty range, the plausibility range was assumed to be 20% of the base value. The PSA was conducted using Monte Carlo simulation (*N* = 1,000 iterations) to assess the impact of changing multiple parameters simultaneously. The results of the DSA and PSA were summarized with tornado diagrams and cost-effectiveness acceptability curves, respectively. Scenario sensitivity analyses were conducted to estimate the break-even prices by varying the prices of TLE strategies.

## Results

### Base-case analysis

Our findings indicate that the TLE400 STR is associated with improved health outcomes, but higher costs compared to both the TLE600 and TLE400 MTR (see Table 2). Over a 10-year period, the average cost for a patient receiving the TLE400 STR was 130733.8 CNY, resulting in a total of 6.45 QALYs gained. In contrast, the costs and total QALYs for the TLE600 and TLE400 MTRs were lower, at 126184.3 CNY (with 6.32 QALYs gained) and 122939.7 CNY (with 6.27 QALYs gained), respectively.

**Table 2:** Cost-effectiveness analysis results for TLE400 single-tablet, TLE400 multiple-tablet, and TLE600 multiple-tablet regimens in China.

Strategies and differences	Total cost (CNY)	QALYs	ICER (CNY/QALY gained)^*^
TLE600 multiple-tablets	126184.3	6.32	-
TLE400 multiple-tablets	122939.7	6.27	-
TLE400 single-tablet	130733.8	6.45	-
Differences (TLE400 STR vs. TLE600 MTR)	4549.5	0.13	35313.6
Differences (TLE400 STR vs. TLE400 MTR)	7794.1	0.19	41706.1
Differences (TLE600 MTR vs. TLE400 MTR)	3244.6	0.06	55893.5

^*^The ICERs may not equal Total cost/QALYs due to rounding in TreeAge Pro 2022.

The incremental cost-effectiveness ratios (ICERs) for the TLE400 STR compared to the TLE600 MTR and the TLE400 MTR were 35313.6 CNY/QALY (equivalent to US$ 5226.4 per QALY gained) and 41706.1 CNY/QALY (equivalent to US$ 6172.5 per QALY gained), respectively. Both ICER values were below the threshold of one-time China’s GDP per capita (85698.0 CNY, equivalent to US$ 12683.3) in 2022, indicating that the TLE400 STR was considered cost-effective in comparison to the other two regimens. Moreover, when comparing the TLE400 MTR with the TLE600 MTR, the ICER for the TLE600 regimen versus the TLE400 regimen was 55893.5 CNY/QALY (equivalent to US$ 8272.2 per QALY gained), which was also below the one-time national-level GDP per capita threshold in 2022. Therefore, the TLE600 MTR was found to be more cost-effective when compared to the TLE400 MTR.

### Sensitivity analyses

**Figure 2:**
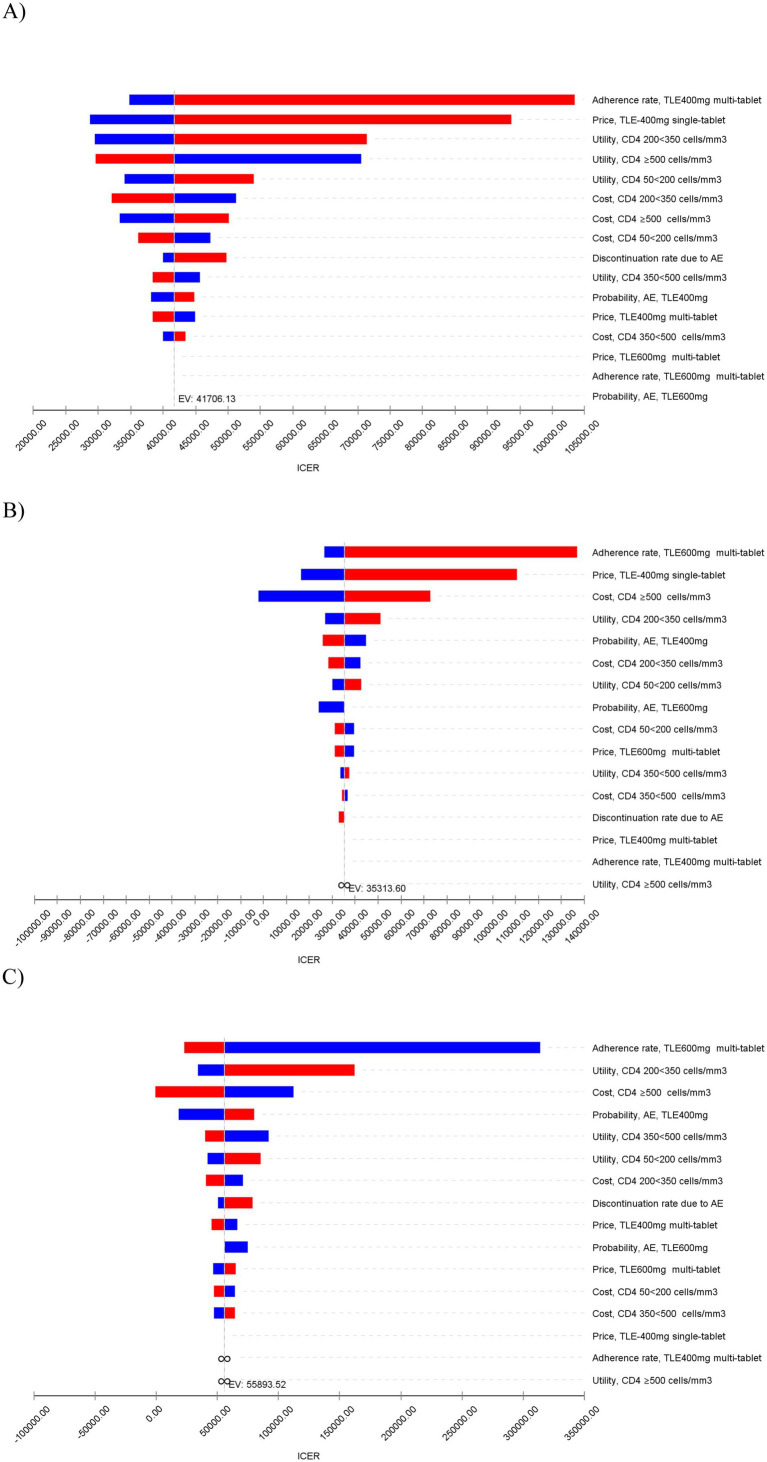
Tornado diagram of deterministic sensitivity analyses for the most influential model parameters on ICER (CNY/QALY gained). **(A)** TLE400 STR vs. TLE400 MTR. **(B)** TLE400 STR vs. TLE600 MTR. **(C)** TLE400 MTR vs. TLE600 MTR.

**Figure 3:**
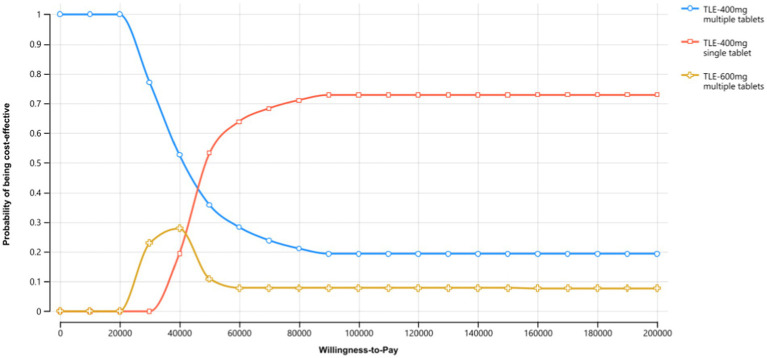
Cost-effectiveness acceptability curves of three TLE strategies.

In the sensitivity analyses, we conducted various assessments to evaluate the robustness of the model results by examining key parameters. In the deterministic sensitivity analyses, the parameters that exhibited the most substantial impact on the results were adherence rates to ART, utilities, the price of TLE400 STR and costs (Figure 2). Alterations in these parameters resulted in variations in the ICERs of the treatment strategies. In the probabilistic sensitivity analysis, which involved Monte Carlo simulation with 1,000 iterations, the TLE400 STR demonstrated a 71.4% probability of being highly cost-effective at the national level when compared to the threshold of one-time GDP per capita in 2022 (Figure 3).

In the price analysis scenario, we explored the impact of varying the prices of the TLE strategies on the ICER values. As the price of the TLE400 STR increased, the ICER values for both comparisons (TLE400 STR vs. TLE400 MTR and TLE400 STR vs. TLE600 MTR) also increased. The break-even prices, at which the ICER values were equal to the one-time national-level GDP per capita in 2022, were determined to be 9.4 CNY per pill for the comparison of TLE400 STR and TLE400 MTR, and 7.8 CNY per pill for the comparison of TLE400 STR and TLE600 MTR. The detailed results and visual representations of the scenario analysis were shown in Appendix Figures 1, 2.

## Discussion

Our study provides valuable insights into the cost-effectiveness of the TLE400 STR compared to the TLE400 MTR and TLE600 MTR for the treatment of HIV-1 infected adults in China. This study is the first to comprehensively analyze all three types of TLE regimens and specifically focuses on the TLE400 STR in the Chinese context. Our findings indicate that the TLE400 STR is a cost-effective option compared with the two MTR strategies in China. The results of our sensitivity analysis also underscore the importance of adherence rates to ART in the economic evaluation model. PSA indicates that the TLE400 STR is likely to be a cost-effective option when considering the uncertainties associated with multiple parameters simultaneously. The TLE400 STR demonstrated higher adherence rates compared to the TLE400 MTR and TLE600 MTR. This can be attributed to the individual benefits of dose reduction, particularly in terms of reducing dose-related toxic effects associated with efavirenz (9, 23). Lower EFV doses have been shown to result in fewer central nervous system adverse events and treatment-related discontinuations, leading to improved self-reported adherence (13, 37). Moreover, the high daily pill burden of multiple-tablet regimens is known to decrease adherence to ART and increase the likelihood of discontinuation (18, 30, 38). In contrast, single-tablet regimens are associated with better adherence rates and non-inferior clinical outcomes. These factors contribute to the higher adherence rates and larger QALYs gained with the TLE400 STR in our study.

Considering the cost-effectiveness analysis, the TLE400 STR is deemed cost-effective despite its slightly higher costs compared to the other two regimens. The ICERs of the TLE400 STR remain below the recommended threshold of one-time China’s GDP per capita, indicating that the regimen offers good value for its cost. Therefore, the TLE400 STR is considered a more valuable treatment option for HIV-1 infected patients in China. Additionally, deterministic sensitivity analysis indicated that adherence rates to ART had the most significant influence on all three strategies. According to existing literature, the number of tablets in a regimen is an important factor affecting adherence rates to ART (18, 30).

Furthermore, our scenario analysis provides insights into the break-even prices for the TLE400 STR based on the recommended threshold of one-time GDP per capita (39). The analysis demonstrates that lowering the prices of TLE strategies, specifically through the reduction of EFV dose, can enhance the cost-effectiveness of the regimens. This has significant implications as it not only benefits patients in terms of improved health outcomes but also alleviates the financial burden on the government and society.

It is important to acknowledge that our analysis has a limited time horizon of 10 years, focusing on short-term outcomes, as long-term data specific to China are not readily available. Future research could address this limitation by investigating the long-term implications and cost-effectiveness of the TLE400 STR over an extended period. Such research would provide a more comprehensive understanding of the economic value of the TLE400 STR and its sustained impact on healthcare outcomes and costs.

### Limitations

While our study provides valuable insights, it is important to acknowledge several limitations. Firstly, the lack of specific data in China required us to reference parameters from studies conducted in other countries and regions. This introduces potential differences in clinical parameters, case fatality rates, and utilities. For example, we acknowledged that using generic utility value sets from China might hold more weight than specific value sets from another country. However, sensitivity analyses conducted in our study indicated no significant impact on our results. Nevertheless, obtaining more representative data from Chinese HIV-infected patients would enhance the robustness of future studies. Secondly, our model did not consider stratification by biological factors, such as body weight. It has been observed that Chinese HIV-infected patients with lower body weight may experience higher efavirenz plasma concentration compared to those with higher body weight (12). Incorporating such stratification into the model would provide a more accurate assessment of the cost-effectiveness of the TLE400 STR for different patient populations. Future studies should aim to collect data specifically for patients with low body weight in China. Thirdly, our model did not account for the impact of treatments associated with first-line treatment failures. This simplification was made to mitigate excessive uncertainties and data limitations. However, it is important to acknowledge that the inclusion of such treatments may provide additional benefits and cost savings associated with the TLE400 STR. Future studies that incorporate comprehensive data on treatment failures would yield a more comprehensive evaluation of the regimen’s cost-effectiveness. Fourthly, we acknowledged that the use of CD4 count as the outcome measure had certain limitations, as many global bodies had since adopted viral load as the measure of clinical outcomes. Overall, while our study provides valuable insights into the cost-effectiveness of the TLE400 STR, it is essential to consider these limitations when interpreting the results. Future research should aim to address these limitations and provide more accurate estimations of the economic value of the TLE400 STR in the context of HIV-1 treatment in China.

## Conclusion

In conclusion, our study demonstrates that the TLE400 STR is a cost-effective treatment strategy for HIV-1 infected adults in China. The TLE400 STR, compared to the TLE400 MTR and TLE600 MTR, offers improved health outcomes despite higher costs, making it economically favorable. The lower efavirenz dose (400 mg) in the STR shows potential advantages in terms of higher adherence rates and better health outcomes. Incorporating the TLE400 STR as the preferred first-line treatment in the National Free AIDS Antiretroviral Drug List, at an optimal price, would provide healthcare professionals and policymakers with an additional option to address the growing burden of HIV in China. However, it is crucial to acknowledge the limitations of our study, including the reliance on data from other countries and the need for more specific data on the Chinese population, particularly for patients with low body weight. Further research, including clinical trials and studies incorporating local data, is warranted to validate our findings and provide more precise estimations of the cost-effectiveness of the TLE400 STR. Overall, the inclusion of this regimen in the national HIV treatment guidelines has the potential to bring significant benefits to individuals living with HIV and the healthcare system in China.

## Data Availability

The original contributions presented in the study are included in the article/Supplementary material, further inquiries can be directed to the corresponding authors.

## Supplementary material

The Supplementary material for this article can befound online at: https://www.frontiersin.org/articles/10.3389/fpubh.2025.1429461/full#supplementary-material
