# Supplementary material for: The cost-effectiveness analysis of single-tablet efavirenz-based regimen among HIV-1 infected adults in China
Source: Front Public Health. 2025 May 8;13:1429461. doi: 10.3389/fpubh.2025.1429461 (PMC12095232; doi:10.3389/fpubh.2025.1429461)
Supplement: Supplementary file 1 [file Table_1.docx]

**Appendices to “The cost-effectiveness analysis of single-tablet efavirenz‑based regimen among HIV-1 infected adults in China”**

**Appendix Figure 1. The price scenario analysis of TLE400 single-tablet and TLE400 multiple-tablet regimen**

9.4

**Appendix Figure 2. The price scenario analysis of TLE400 single-tablet and TLE600 multiple-tablet regimen**
